# Can industry-education integration enhance enterprise employee safety and health levels?

**DOI:** 10.3389/fpubh.2025.1607953

**Published:** 2025-07-30

**Authors:** Huiling Chen, Chengcheng Zhu, Zhao Cheng, Jie Feng, Boya Zhao

**Affiliations:** ^1^College of Accounting (College of Wealth Management), Ningbo University of Finance and Economics, Zhejiang, China; ^2^School of Management Science and Real Estate, Chongqing University, Chongqing, China; ^3^School of Public Health, Peking University, Beijing, China; ^4^Department of Social Work, The Chinese University of HongKong, Hong Kong, Hong Kong SAR, China

**Keywords:** industry-education integration, employee safety and health, internal control, media oversight, difference-in-differences model

## Abstract

**Introduction:**

Harmonious relationships between enterprises and employees are key to sustainable enterprise development. Therefore, the study explores whether industry-education integration can enhance enterprise employee safety and health levels, in order to alleviate enterprise labor-management issues.

**Methods:**

Based on panel data from Chinese A-share listed enterprises between 2012 and 2021, this study employs a quasi-natural experiment using China’s pilot program for industry-education integrated enterprises. A difference-in-differences model is constructed to examine how this novel employee training model affects enterprise employee safety and health levels.

**Results:**

This study finds that, first, industry-education integration promotes an increase in enterprise employee safety and health. Second, the mechanism behind this effect lies in enhancing enterprise internal control systems and strengthening media oversight. Third, this effect is more pronounced in state-owned enterprises and enterprises with stronger management capabilities. Fourth, industry-education integration also drives improvements in enterprise technological innovation.

**Discussion:**

This study provides evidence of the positive impact of this new training model on enterprise development and offers corresponding policy recommendations.

## 1 Introduction

The global economy is currently experiencing a period of slowing growth and industrial restructuring, making employment issues more complex. According to the *World Employment and Social Outlook: Trends2025*, global economic growth is slowing down, making it more difficult for the labor market to fully recover. At the same time, the global unemployment rate was 5.0% in 2024, and the labor force participation rate is declining. Of greater concern is the fact that The estimated number of people who wish to find work but are unable to do so is 402 million, including 186 million unemployed persons, 137 million workers who are temporarily unemployable, and 79 million discouraged workers who have given up looking for work. This highlights the persistent severity of global employment challenges, with job opportunities remaining scarce.

The employment issue in the Asia-Pacific region is particularly concerning. According to the *World Employment and Social Outlook* released by the International Labour Organization (ILO, see Table S1), the Asia-Pacific region faces problems such as huge employment gaps, gender gaps and informal employment. These labor-capital conflicts have led to frequent collective incidents, disrupted business operations and threatened social stability. What is more serious is that the health and safety of employees in the Asia-Pacific region are exposed to greater risks (1, 2). According to the *Promoting a Safe and Healthy Working Environment* released by the ILO, nearly 3 million people worldwide die from work-related accidents and diseases, with the highest proportion in Asia and the Pacific region. Accounting for 63% of the global work-related mortality rate.

From the perspective of labor economics, stable employment relationships are a prerequisite for encouraging enterprises to invest in human capital and improving technological production efficiency (3, 4). Therefore, the health and safety of employees are of great significance to business operations, economic development and social stability. On a micro-level, employee safety and health can alleviate employees’ feelings of insecurity, thereby influencing their work attitudes and behaviors (5–7). At the macro level, employee safety and health helps to reduce labor-management conflicts and mitigate employment fluctuations, thus promoting economic development and maintaining social stability.

In recent years, many countries enacted laws and regulations to improve the level of employee safety and health for workers (8–10). For example, in 2015, the Netherlands introduced the *Work and Security Act*, updating dismissal regulations, and in 2020, it enacted the *Balanced Labour Market Act* (11). However, COVID-19 is ringing alarm bells globally about the dangers posed to employee health and safety (12, 13). How to maintain and improve employee health and safety in the long term remains a concern.

This paper focuses on how a new employee training model in Chinese enterprises affects employee safety and health levels for three main reasons. First, this paper focuses on China because employee health and safety issues become particularly pronounced there. According to China’s *Ministry of Human Resources and Social Security*, in 2023, there were 28,882 labor and social security dispute cases, reflecting an approximate 16% year-on-year increase. Meanwhile, China has performed well in responding to the negative impact of COVID-19 on the health of its employees (14). This complex situation provides us with an opportunity to explore how to ensure the health and safety level of employees. But, academic discussions on this topic remain insufficient, particularly in terms of empirical research.

Second, in 2017, China introduced a new employee training model known as industry-education integration. This model is not only a key institutional design for attracting talent to enterprises but also an essential measure for retaining and developing talent. With the pilot implementation of industry-education integration, highly educated and innovative talent increasingly flowed into the pilot enterprises, injecting fresh vitality from universities and research institutes into these enterprises. This also heightened employees’ demand for employee safety and health and elevated their standing in dialogues with enterprise management. As a result, industry-education integration can influence enterprises from the bottom up, encouraging more employees to actively express their demands and contribute to the improvement of enterprise governance mechanisms, thus enhancing employee safety and health levels.

Third, China’s industry-education integration pilot program provides an excellent quasi-natural experiment. In 2019, China issued the *National Pilot Implementation Plan for Industry-Education Integration*, mandating the first batch of national industry-education integrated cities and enterprises. This allows us to treat pilot enterprises as the experimental group and non-pilot enterprises as the control group to construct a difference-in-differences (DID) model, enabling a more accurate identification of the micro-level impacts of this new employee training model.

Based on this, this paper uses panel data from Chinese A-share listed enterprises from 2012 to 2021, with the construction of national industry-education integrated enterprises serving as a quasi-natural experiment. Through the construction of a DID model, this paper explores whether industry-education integration can enhance employee safety and health levels. Our findings indicate that industry-education integrated enterprises significantly improve the level of employee safety and health, primarily through enhancing internal and external enterprise governance mechanisms. Moreover, this effect is more pronounced in state-owned enterprises and enterprises with stronger management capabilities.

This study makes three key marginal contributions. First, it uses the pilot construction of industry-education integrated enterprises as a quasi-natural experiment and applies a DID model to examine its impact on enterprises. Existing research addressed the effects of university-industry collaboration on enterprises (15, 16). However, compared to university-industry collaboration, industry-education integration is not a one-off project partnership but rather involves the deep integration of university students into enterprises as employees. This represents an innovative employee training model that can exert long-lasting and profound effects on enterprises. Our study provides empirical evidence of the positive micro-level impacts of industry-education integration.

Second, based on human capital theory and corporate social responsibility theory, this study confirms the positive effect of industry-education integration on employee safety and health. While existing research extensively explores how enterprise systems and managerial characteristics influence employee safety and health, this paper demonstrates how this new employee training model, industry-education integration, affects enterprise employment systems. Additionally, this paper explores the mechanisms and boundary conditions under which industry-education integration exerts its influence, helping policymakers better understand the theoretical logic behind its effects.

Finally, based on a series of research findings, this study offers specific policy recommendations for advancing and improving the industry-education integration system. For higher education, industry-education integration is a transformative initiative that shifts university students from classroom-based theoretical learning to enterprise production practices. For enterprises, industry-education integration is not only a new training model to comprehensively enhance employee capabilities and competencies but also a critical measure for improving internal governance and promoting sustainable development. This research provides valuable insights for policymakers and enterprise management alike.

## 2 Literature review and hypothesis development

### 2.1 Literature review

#### 2.1.1 Study on industry-education integration

As the structural mismatch of human capital becomes increasingly prominent, industry-education integration has garnered growing attention from both academia and policymakers as an institutional approach to supply-side reform. This mechanism aims to promote deep collaboration between corporations and higher education institutions, thereby optimizing the allocation of educational resources, enhancing the quality of talent cultivation, and, more broadly, supporting labor market restructuring and the implementation of corporations’ long-term development strategies.

Existing research has primarily approached industry education integration from the perspective of educational supply, focusing on its role in enhancing employability. Burchert et al. (17), drawing on vocational education reform cases in Germany and the Netherlands, argued corporations play a pivotal role in the dual education system. Their active engagement in vocational training effectively reduces employee turnover and fosters long term stability in employment relationships. Grimshaw and Rubery (18), based on the context of human resource management within inter organizational networks in the United Kingdom, revealed the profound impact of networked structures on job adaptability and employment stability. Building on these findings, some scholars have shifted attention to the individual level, examining the influence of industry education integration on talent development and employment adaptability. For example, Plessis et al. (19) emphasized that close collaboration between universities and industries plays a critical role in improving graduate employability in the post COVID-19 era, and called for greater attention to aligning education with labor market demands and fostering contextual competencies.

Recently, scholars have begun to examine the spillover effects of industry education integration on corporate governance and innovation capacity. Using European Union-level micro-level data, Rosário et al. (20) provide empirical evidence that the mechanism markedly increases corporate research and development investment and patent output, thereby continuously strengthening organizational innovation capability. Focusing on Morocco as an emerging market context (21), find that collaboration between corporations and universities reinforces internal oversight and governance structures, which in turn renders ESG disclosure more normative and transparent. A growing body of work also situates industry education integration within the framework of sustainable development and institutional governance. For example, Cai et al. (22), drawing on a sample of corporations listed in the S&P500, confirm that close integration of talent investment with governance mechanisms improves overall ESG performance, with especially pronounced effects in human resource strategy and environmental information disclosure. As the role of industry education integration in organizational performance, governance transparency, and sustainable objectives becomes increasingly prominent, academic attention has gradually shifted from localized coordination mechanisms to a more systematic understanding of its macro institutional logic and knowledge production structure. The triple helix theory emphasizes that the triadic interaction among universities, corporations, and governments constitutes a core mechanism for structural innovation and resource optimization in knowledge-based societies. This perspective provides a robust theoretical foundation for industry education integration. As Abdullah and Hridoy (23) point out, industry education integration is not only a catalyst for innovation but also an effective pathway to enhance research quality and practical relevance, serving as a critical bridge between theory and practice.

In summary, current research has revealed the multifaceted benefits of industry education integration from various perspectives, yet limitations remain in both theoretical construction and empirical identification. First, a substantial body of literature continues to focus on the macro effects of industry education integration on corporate performance or innovation output, while overlooking critical dimensions at the individual employee level, such as workplace safety, mental health, and occupational disease prevention. Second, most existing analyses are based on correlational frameworks, and the methodologies employed have not effectively addressed endogeneity issues, thereby weakening the validity of causal inference. Finally, corporate heterogeneity has not been adequately explored. In particular, significant differences may exist in policy absorption capacity and institutional response mechanisms between corporations of different ownership types and managerial capacities, which have yet to be thoroughly examined.

#### 2.1.2 Study on employment of employees

As enterprises increasingly emphasize high-quality development, employment security has become closely tied to employee health and safety. Scholars have long focused on employee benefits as a key dimension of protection and motivation. De la Torre-Ruiz et al. (24) found flexible benefit programs significantly improve employee satisfaction. Perrault and Hildenbrand (25) observed that most employees are aware of only a fraction of their benefits, highlighting the need for improved internal communication. Immediately after that, Cordón-Pozo (26), using Spanish survey data, confirmed effective communication enhances satisfaction with benefits.

Beyond motivation, benefits have substantial health implications. Adikes et al. (27) emphasized that employee benefits are vital for improving the health of low-income workers. Schauer et al. (28) found that well-structured benefits programs help employees manage their own health more effectively. Messersmith et al. (29) showed that provisions such as health insurance, flexible schedules, and paid leave increase firm survival rates. Bao (30), through a DID model, found paid family leave programs improve profitability in innovative corporations.

Some scholars have investigated child labor, which directly affects health and safety. Dumas (31) found improved market conditions reduce child labor in Madagascar. Fumagalli and Martin (32), using a DID model, showed that Mozambique’s adult labor program lowered child labor by reducing adult labor costs and increasing productivity. Le and Homel (33) found child labor in Vietnam significantly harmed academic performance, particularly for girls. Pirkle et al. (34), using data from Ghana and Côte d’ Ivoire, linked hazardous child labor conditions to higher injury risks and increased school repetition.

Research on workplace safety has gained momentum. Masso (35), through a case study in Estonia, revealed that practical safety management approaches are more influential than abstract safety culture in promoting participation in occupational health systems. Mullen et al. (36) found that transformational leadership centered on safety strengthens safety compliance, participation, and attitudes. Bayram (37) confirmed that safety training, knowledge, and motivation significantly improve safety-related productivity. Liu et al. (38) discovered that spiritual leadership enhances employees’ sense of mission and thus improves their safety performance.

Despite these contributions, current research faces several limitations. First, there is a lack of attention to employee health and safety in Chinese enterprises, where such concerns are often severe. Second, most studies isolate specific aspects of employment security, even though factors such as insurance costs (39), labor disputes (40), and gender discrimination (41) may interact to shape safety outcomes. A comprehensive index for evaluating employee safety and health within enterprises is therefore necessary. Third, existing literature primarily adopts a top-down perspective, focusing on enterprise strategies and policy-level drivers. There remains limited understanding of how safety and health outcomes can be improved through bottom-up mechanisms initiated by employees themselves.

### 2.2 Policy interpretation

In 2017, China emphasized the need to deepen industry-education integration in the report of the *19th National Congress*. That same year, the *Several Opinions on Deepening Industry-Education Integration* (hereinafter referred to as “the *Opinions*”) were issued. The aim was to comprehensively enhance the quality of education and employment, while promoting economic transformation and upgrading. The *Opinions* provided enterprises with a more pragmatic and efficient platform for talent cultivation, allowing them to participate directly in the talent development process. This helps ensure that the graduates produced by educational institutions better meet the needs of enterprises for innovative talent. At the same time, enterprises can secure potential talent in advance, shorten the onboarding process for new employees, and improve hiring success rates.

The introduction of the *Opinions* also offered an effective solution for enterprises to address challenges related to employee security. Consequently, in 2019, China launched the *National Pilot Implementation Plan for Industry-Education Integration* (hereinafter referred to as “the *Implementation Plan*”) to deeply implement industry-education integration. The goal was to promote the organic alignment of the education, talent, industrial, and innovation chains by creating a platform for resource sharing and complementary advantages, enabling enterprises to gain sustained talent and intellectual support in market competition. The *Implementation Plan* represents the concrete execution of the *Opinions* and required relevant regions to recommend pilot enterprises by November 20, 2019. China released a list of industry-education integration pilot enterprises, including National Nuclear Corporation, Aerospace Science and Industry Corporation, and State Grid Corporation, among 63 other enterprises.

China’s pilot reform of industry-education integration has four key features. First, the pilot enterprises are spread across 25 provinces, covering the eastern, central, and western regions, providing a degree of randomness to the experimental design. Second, the local development and education departments are required to leverage the demonstration role of national industry-education integrated enterprises and pilot cities. They must establish a new reform pathway and mechanism focusing on cities as nodes, industries as leverage points, and enterprises as focal points. The primary tasks include promoting talent development reform, constructing innovative major platforms, and exploring institutional and mechanism innovations. Third, pilot cities and enterprises must refine their work tasks, carefully implement policy requirements, and ensure the achievement of pilot objectives within the set timeframe. Fourth, relevant national departments must enhance coordination and oversight, organizing timely evaluations of the implementation progress.

### 2.3 Hypothesis development

Human capital, as a factor of production, has long attracted the attention of scholars for its impact on enterprises. According to human capital theory, the quality and capabilities of individuals profoundly influence the level and quality of economic development, often exceeding the impact of capital and resources. In an enterprise’s production and operations, specialized vocational training is used to enhance employees’ abilities to adapt to complex challenges, aligning their personal skills with the enterprise’s talent needs. Moreover, specialized training helps stabilize the relationship between employees and the enterprise. Based on social exchange theory, when an enterprise invests in employee training or skill development, it signals to employees that they are valued, which motivates them to contribute more steadily and actively to the enterprise’s growth (42).

At the same time, with the rapid development of the knowledge economy and significant changes in business models, employment relationships have become increasingly critical to enterprise success. In traditional employment relationships, employees typically have little voice in major enterprise decisions (43). As a result, the level of importance placed on employees is often determined by top-down decisions from management. This suggests that the extent of employee training and other employment protections is contingent on the social responsibility of the management team. Although management may arrange professional training due to its positive effects on the enterprise, employees are rarely given more say in the employment or training process.

According to corporate social responsibility theory, enterprises should not only safeguard the interests of shareholders but also consider other stakeholders, including employees, suppliers, and potential interest groups, thereby contributing to the broader progress of society. As labor relations become increasingly strained, improving employee safety and health levels has become an important component of enterprise social responsibility (44). It is not only crucial for the stability of internal management but also has a profound impact on the enterprise’s social reputation and image.

Industry-education integration is an innovative employee training model that creates a platform for two-way communication between management and employees. On the one hand, industry-education integration allows university students to become potential employees of enterprises, enabling them to seamlessly integrate their professional skills with career planning, thus forming realistic career expectations. To reduce the risk of losing these highly skilled potential employees, enterprises must increase employment security to enhance their attractiveness.

On the other hand, with the advancement of societal values, more and more university students place greater emphasis on equality, respect, and humanistic care in their employment. Existing literature suggests that university students, compared to others, place higher importance on enterprise social responsibility (45). Through the platform of industry-education integration, students can combine theoretical learning with practical experience, improving their personal skills while securing future employment protections. In this way, industry-education integration offers a new channel for enterprises and management to listen to employees’ demands.

Based on this, this paper proposes:

*Hypothesis 1: Industry-education integration promotes higher levels of employee safety and health.*

From the perspective of internal governance, drawing on the resource based view and stakeholder theory, industry-education integration serves as a strategic resource that enhances internal governance by optimizing human capital and organizational processes.

First, industry-education integration acts as an informational bridge between enterprises and universities, mitigating information asymmetry. Industry-education integration, as a novel employee training model, provides potential employees from universities the opportunity to engage in dialogue with management. The resultant transparency in operations fosters monitoring efficiency, a core dimension of internal control (46).

Second, from a human resources management perspective, enterprises can specify recruitment requirements for these potential employees prior to their employment, thus fostering talent that is tailored to the enterprise needs. As a result, industry-education integration reduces the adaptation period and the risk of turnover among new hires (47). The human capital specificity enhances control effectiveness by ensuring employees’ adherence to organizational routines and internal control.

Third, enterprises can leverage the research and development advantages of universities and research institutions through industry-education integration to jointly analyze and mitigate business risks, thereby enhancing their capacity to identify and manage risks through improved internal controls. Such knowledge recombination augments firms’ dynamic capabilities, enabling proactive internal control adjustments, a critical factor for risk management.

Strengthened internal controls institutionalize ethical practices (48). Resource based view posits that such controls create isolating mechanisms to safeguard employee welfare, translating governance improvements into measurable safety and health outcomes. Therefore, strengthened internal controls facilitate the fulfillment of enterprise social responsibility (49), which positively impacts employee safety and health.

Based on this, this paper proposes:

*Hypothesis 2: Industry-education integration enhances internal controls, which in turn promotes higher levels of employee safety and health.*

From the perspective of external governance, industry-education integration enhances media oversight, which in turn improves employee health and safety. Media coverage has a profound effect on corporate behavior. First, it alters stakeholders’ perceptions of corporate leadership (50), creating career concerns that incentivize compliance. Second, it generates reputational penalties that impose measurable financial costs (51, 52).

The unique context of IEI creates institutional conditions that heighten media oversight through three mechanisms. First, in response to government policies, local governments are introducing supportive policies for industry-education integrated enterprises, including subsidies. Government support for IEI enterprises generates newsworthy signaling events (53). With government involvement, industry-education integration naturally draws media attention and oversight, thereby fostering collaborative supervision by both government and media (54).

Second, as a nationally promoted training model, industry-education integration presents both challenges and opportunities for pilot enterprises. As nationally mandated pilot programs, IEI enterprises face heightened visibility. Pilot enterprises can proactively engage with media to showcase their training outcomes, thereby building a positive public image (55). In the process of media coverage, the media also serves as a supervisory force.

Third, since the participants in industry-education integration are university students, they gain firsthand insight into the enterprise’s real operations by combining classroom learning with enterprise practice. Student participants act as boundary spanners (56), whose social media sharing behavior creates organic visibility. As media technology evolves, these students are eager to share their learning and work experiences on social media platforms, significantly increasing the exposure of pilot enterprises and attracting the attention and supervision of official media.

External oversight, especially media scrutiny, plays a key role in holding enterprises accountable (57), urging them to enhance employee safety and health levels. Employee safety and health are the basic rights that employees should enjoy. It is an illegal act for a company to fail to guarantee the basic rights of its employees. If this act is disclosed, it will definitely cause losses to the company, such as penalties from regulatory authorities and negative evaluations from the market. Additionally, external supervision complements internal control mechanisms by addressing their potential shortcomings (58). Therefore, the implementation of industry-education integration strengthens external media supervision, thereby promoting employee safety and health levels.

Based on this, this paper proposes:

*Hypothesis 3: Industry-education integration attracts media oversight, which in turn promotes higher levels of employee safety and health.*

## 3 Empirical design

### 3.1 Model and variables

This study employs a multi-period DID model with multidimensional fixed effects to assess the impact of the pilot construction of industry-education integrated enterprises. By establishing both experimental and control groups, this paper treats the pilot construction of industry-education integrated enterprises as a quasi-natural experiment. This approach effectively reduces “noise” in the research and mitigates issues related to endogeneity. The baseline regression model is as follows:


(1) $$\mathrm{Saf}e_{i,t}=\alpha +{\text{βTreat}}_{i}\times {\text{Time}}_{t}+\text{γControl}s_{i,t}+\delta +\mu +\varepsilon$$

This paper employs a mediation effect model to examine the mechanisms in this study. The model is as follows:


(2) $$\text{Machin}e_{i,t}=\alpha +{\text{βTreat}}_{i}\times {\text{Time}}_{t}+\text{γControl}s_{i,t}+\delta +\mu +\varepsilon$$

(3) $$\begin{array}{l} \mathrm{Saf}e_{i,t}=\alpha +{\text{βTreat}}_{i}\times {\text{Time}}_{t}+\text{θMachin}e_{i,t}+\text{γControl}s_{i,t}\\ +\delta +\mu +\varepsilon \end{array}$$

In the above model, i represents the enterprise, t represents the year, δ denotes the individual (enterprise) fixed effects, μ represents the time (year) fixed effects, and ε denotes the error term.

$\mathrm{Saf}e_{i,t}$ is the dependent variable, representing employee safety and health. In this study, this paper obtains employee security scores from the *Enterprises’ Contribution to Common Prosperity Research Database* within the CSMAR collaborative database, which this paper uses as *Safe1*. This database was jointly developed by CSMAR and the *East China Normal University*’s research team on sustainable development and corporate social responsibility. The employee security system includes seven indicators that comprehensively reflect the level of employee safety and health within enterprises (Table 1).

**Table 1:** Measurement system for safeguard.

Indicator	Measurement
Legal Employment	If the enterprise has issued documents prohibiting the use of child labor or disclosed in CSR reports that child labor is not used, it is scored as 1, otherwise 0.
Investment in Safety Production	Investment in safety production/operating income
Safety Production Level	If the enterprise has experienced a major safety incident, general safety incident, or occupational accident, it is scored as 1, 0.8, or 0.6, respectively. If multiple incidents have occurred, scores will be cumulative. If no incident has been disclosed or occurred, it is scored as 0.
Occupational Health Protection	If the enterprise has disclosed investment in employee health checks, occupational disease treatment, or occupational health protection funds, conducted occupational safety training, or disclosed the absence of occupational health accidents, it is scored as 1, otherwise 0.
Employee Social Security Contribution Ratio	Social security expenses/total employee wages
Commercial Insurance	Commercial insurance investment/operating income
Protection of Employee Rights and Benefits	Number of employee-related lawsuits/total number of employees

${\text{Treat}}_{i}\times {\text{Time}}_{t}$ is the key explanatory variable in this study, representing the policy variable for the pilot construction of industry-education integrated enterprises, constructed through the DID model. Here, ${\text{Treat}}_{i}$ is a dummy variable, where treat equals 1 if the enterprise is selected as an industry-education integrated enterprise, and 0 otherwise. ${\text{Time}}_{t}$ is also a dummy variable, where time equals 1 for the period after the implementation of industry-education integration in 2019 and onwards, and 0 otherwise. The interaction term, treat×time, represents the net difference between pilot and non-pilot enterprises before and after the implementation of industry-education integration.

It is important to clarify that although the official list of industry-education integrated pilot enterprises was not released until 2021, the various regions had already recommended enterprises to the *National Development and Reform Commission and the Ministry of Education* in 2019. Therefore, in this study, the time variable uses 2019 as the cutoff, with time equal to 1 for 2019 and beyond, and 0 otherwise.

$\text{Machin}e_{i,t}$ refers to the mechanism variables, which include *IC* and *Media*. *IC* represents internal control within the enterprise, for which this paper uses the internal control index from the DIB database as a proxy variable, applying a logarithmic transformation. *Media* represents media supervision, for which this paper uses the total number of newspaper reports mentioning the enterprise in financial news as a proxy variable, also applying a logarithmic transformation.

$\text{Control}s_{i,t}$ refers to a set of control variables selected for this study, including factors such as the financial status of the enterprise, enterprise governance structure, and regional development level. The specific definitions of these variables are provided in Table 2.

**Table 2:** Definitions of control variables.

Variable symbol	Variable definition	Variable measurement
*size*	Enterprise size	Ln (Total assets)
*ltime*	Years listed	Ln (Current year-listing year)
*roa*	Return on assets	Net profit/total assets
*lev*	Leverage ratio	Total liabilities/total assets
*kz*	Financing constraint	KZ index
*board*	Board size	Ln (Number of board members)
*top1*	Ownership concentration	Shareholding ratio of the largest shareholder
*wage*	Executive compensation	Total executive compensation/total assets
*holding*	Executive shareholding	Executive shareholding ratio
*grp*	Regional GDP	Ln (Regional GDP)
*industry*	Industrial structure	Secondary industry output value/regional total output value

### 3.2 Sample and data

This study selects A-share listed enterprises in China from 2012 to 2021 as the initial research sample. Based on the *List of National Industry-Education Integrated Enterprises*, data was collected from 52 A-share listed enterprises among the 63 pilot enterprises.

The following sample screening steps were conducted. First, this paper excluded samples from the banking and insurance industries. The industries to which the remaining samples belong are shown in Table S2. Second, this paper removed enterprises with a leverage ratio greater than 1, indicating bankruptcy. Third, this paper excluded enterprises that had been listed for 1 year or less and those with only one observation period. Fourth, this paper removed samples with missing data on employee safety and health. After these adjustments, the final sample consists of 3,409 enterprises, with a total of 25,356 observations. Among these, 45 are pilot enterprises.

In this study, data on employee safety and health is sourced from the *Enterprises’ Contribution to Common Prosperity Research Database*, jointly developed by CSMAR and *East China Normal University*. The internal control index comes from the DIB database, and regional economic data is obtained from the China Statistical Yearbook. Other enterprise-level data is collected from the CSMAR database.

Descriptive statistics for the sample data are shown in Table 3. The mean value of *Safe1* is 51.301, with a standard deviation of 3.443, indicating that employee safety and health in Chinese enterprises is relatively low and that there are certain disparities across enterprises. The mean value of *Treat* is 0.015, indicating that industry-education integrated enterprises account for 1.5% of the sample, suggesting that the number of pilot enterprises is relatively small and that further promotion is needed. Descriptive statistics for other variables are not discussed in detail.

**Table 3:** Descriptive statistics.

Variable	Obs	Mean	Std. dev.	Min	Max
*Safe1*	25,356	51.30	3.44	27.31	84.00
*Treat*	25,356	0.02	0.12	0.00	1.00
*Time*	25,356	0.37	0.48	0.00	1.00
*IC*	25,356	6.31	1.02	0.00	6.89
*Media*	25,356	3.14	1.36	0.00	9.27
*size*	25,356	22.31	1.33	14.94	28.64
*ltime*	25,356	2.05	0.91	0.00	3.43
*roa*	25,356	0.04	0.08	−3.99	0.79
*lev*	25,356	0.43	0.20	0.01	0.10
*kz*	25,356	0.90	2.37	−11.33	13.66
*board*	25,356	2.13	0.20	1.10	2.89
*top1*	25,356	0.34	0.15	0.00	0.90
*wage*	25,356	0.00	0.00	0.00	0.26
*holding*	25,356	0.13	0.19	0.00	0.90
*gdp*	25,356	10.57	0.76	6.57	11.77
*industry*	25,356	0.40	0.09	0.16	0.59

## 4 Empirical results

### 4.1 Parallel trend test

A prerequisite for applying the DID model is that the parallel trend assumption must hold, meaning that before the policy intervention, there should be no significant differences in the dependent variable between the treatment group and the control group. To verify this, following the event study approach, this paper constructs the following model to examine the dynamic effects (59).


(4) $${\text{Safe}}_{i,t}=\alpha +\sum_{t=2012}^{2021}\beta_{t}\beta {\text{Treat}}_{i}\times T_{t}+\gamma {\text{Controls}}_{i,t}+\delta +\mu +\varepsilon$$

**Figure 1:**
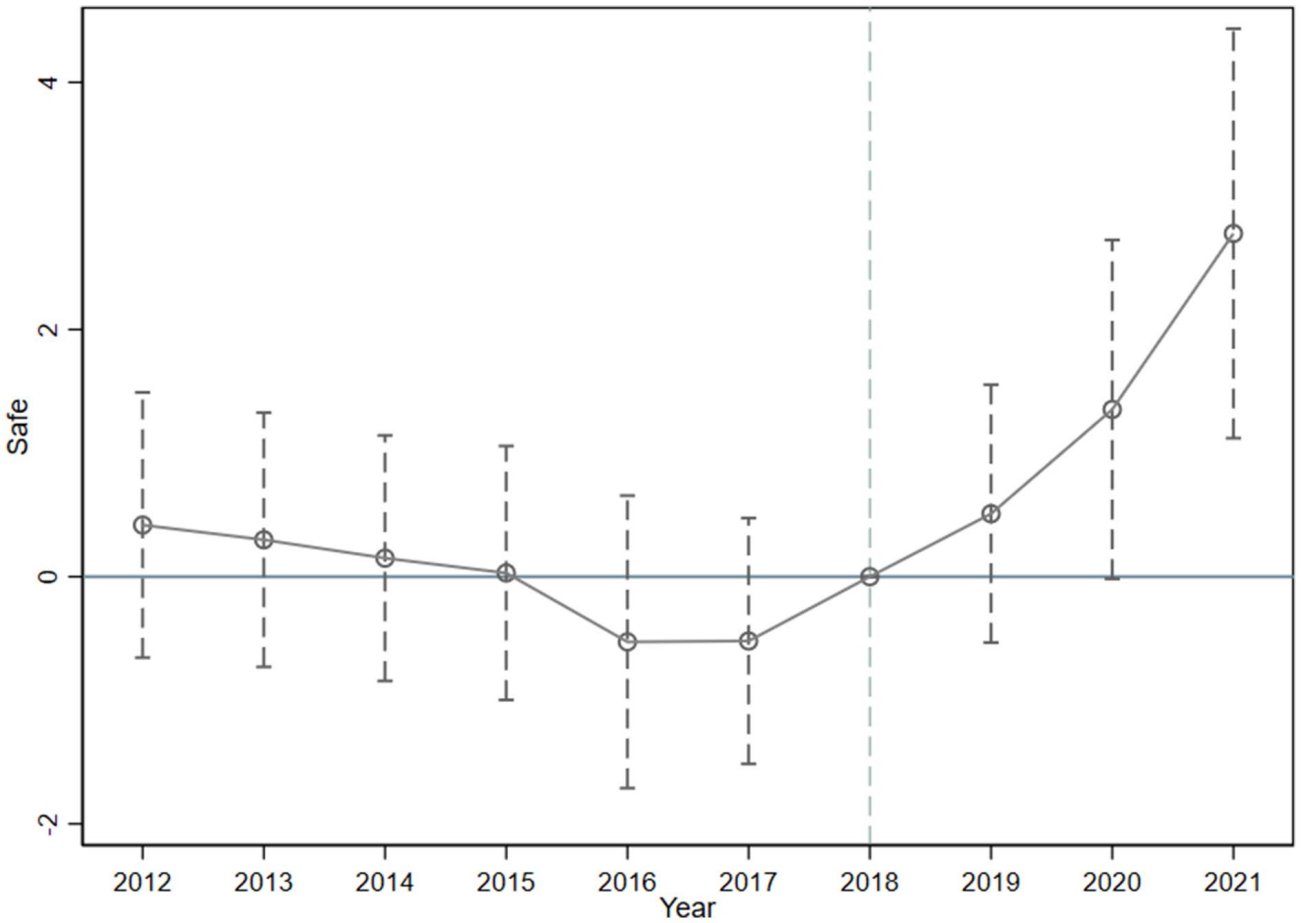
Dynamic effects test.

In this model, *T_t_* represents a series of dummy variables indicating the year, while other elements remain consistent with Model (1). Figure 1 illustrates the results of the dynamic effects test based on Equation 4, with the year prior to the policy intervention (2018) serving as the baseline (60). The hollow points represent regression coefficients, and the dashed lines indicate the 95% confidence intervals

As shown in Figure 1, prior to the pilot construction of industry-education integrated enterprises, the confidence intervals of the regression coefficients include zero, indicating no significant difference in employee security between the pilot and non-pilot enterprises, thus satisfying the parallel trend assumption. Additionally, from 2019 onwards, the coefficient values steadily increase and become significantly greater than zero by 2021, indicating a significant improvement in employee security in pilot enterprises following the implementation of industry-education integration.

### 4.2 Baseline regression results

Table 4 reports the results of the baseline regression based on Equation 1. As shown in Column (1), after controlling for individual fixed effects and time fixed effects, the coefficient of the interaction term (*Treat × Time*) is 1.747, which is significant at the 0.01 level. As shown in Column (2), after further controlling for the control variables, the coefficient of the interaction term (*Treat × Time*) is 1.588, and it remains significant at the 0.01 level.

**Table 4:** Baseline regression results.

	(1) *Safe1*	(2) *Safe1*
Treat × Time	1.75^***^ (0.59)	1.59^***^ (0.59)
*size*		0.26^***^ (0.06)
*ltime*		−0.42^***^ (0.06)
*roa*		0.52^**^ (0.21)
*lev*		−0.29 (0.21)
*kz*		0.01 (0.01)
*board*		−0.36^*^ (0.19)
*top1*		0.06 (0.34)
*wage*		8.46^**^ (3.68)
*holding*		0.75^***^ (0.21)
*grp*		0.48^*^ (0.26)
*industry*		1.27 (1.11)
_cons	51.29^***^ (0.02)	41.52^***^ (2.97)
Individual FE	Yes	Yes
Year FE	Yes	Yes
Observations	25,356	25,356
*R* ^2^	0.54	0.54

***, **, and * indicate significance levels at 0.01, 0.05, and 0.1, respectively. Robust standard errors are reported in parentheses. The remaining tables follow the same note.

These regression results indicate that, compared to non-pilot enterprises, the employee security level in pilot enterprises improved by approximately 1.588 points after the implementation of industry-education integration. Therefore, this suggests that industry-education integration significantly enhances employee security, supporting Hypothesis 1.

### 4.3 Robustness tests

#### 4.3.1 Placebo test

To eliminate the interference from unobservable factors such as region or industry, a placebo test is conducted. This paper performs 500 iterations by randomly selecting 52 enterprises (equal to the number of enterprises in the experimental group) from the sample and treating them as a pseudo-experimental group. If the pseudo-experimental group fails to produce similar results, it would confirm the reliability of our research findings.

**Figure 2:**
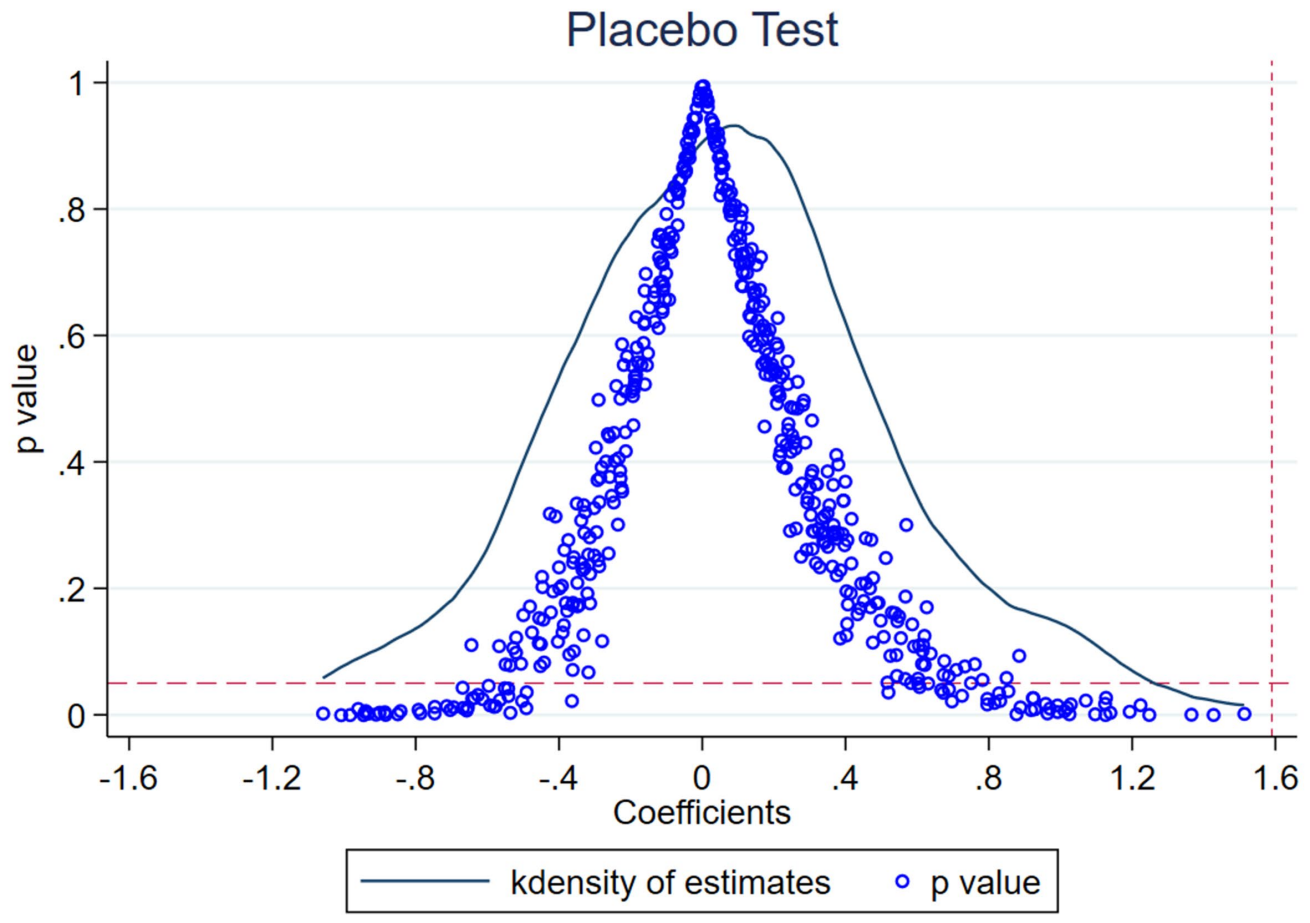
Placebo test.

Figure 2 illustrates the results of the placebo test. It is observed that the majority of the randomly sampled results are insignificant, while the actual regression result is an outlier. Therefore, the research conclusions are minimally affected by these potential unobservable factors.

#### 4.3.2 PSM-DID test

The selection of industry-education integrated enterprises takes into account various factors, such as enterprise development and regional economic conditions, meaning the choice of the experimental group is not entirely random. To eliminate the potential influence of factors affecting the selection of enterprises for the industry-education integration pilot program, this study conducts a regression test after performing propensity score matching (PSM) on the sample (61).

**Figure 3:**
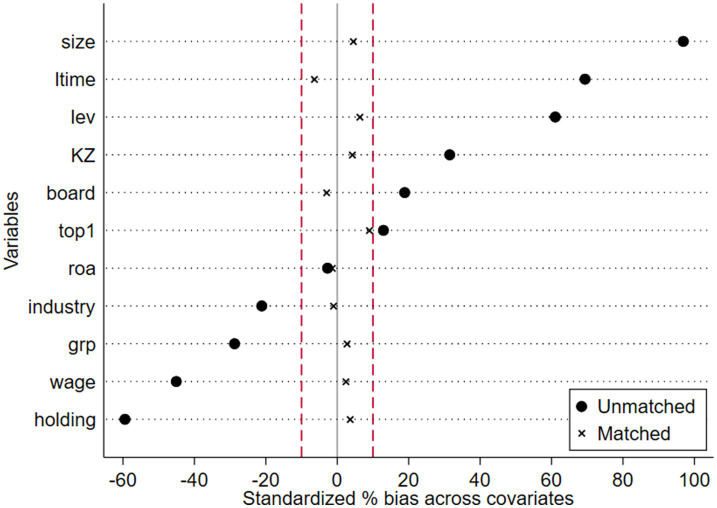
Balance test.

Specifically, all control variables are selected as covariates, and the propensity scores are calculated. Then, based on the propensity scores, 1:1 nearest neighbor matching is conducted. As shown in Figure 3, after PSM matching, the differences in the group means of all variables are insignificant, indicating that the PSM matching is effective.

As shown in Column (1) of Table 5, the number of observations after PSM matching is 454. The coefficient of the interaction term (*Treat × Time*) is 4.189, which is significant at the 0.01 level, demonstrating the robustness of the research results.

**Table 5:** Robustness tests

	PSM-DID	Controlling for other policy interference		Alternative measurement	Other robustness tests		
	(1)	(2)	(3)	(4)	(5)	(6)	(7)
*Treat × Time*	4.19^***^	1.58^***^	1.58^***^	3.15^***^	2.02^***^	1.59^**^	1.16^**^
	(1.26)	(0.59)	(0.58)	(1.11)	(0.77)	(0.81)	(0.49)
*Bigdata*		0.07					
		(0.08)					
*Digital*			0.07^**^				
			(0.03)				
_cons	31.90	41.61^***^	41.91^***^	10.21	38.52^***^	41.52^***^	45.02^***^
	(27.26)	(2.97)	(2.98)	(11.76)	(3.23)	(4.01)	(2.82)
Controls	Yes	Yes	Yes	Yes	Yes	Yes	Yes
Individual FE	Yes	Yes	Yes	Yes	Yes	Yes	Yes
Year FE	Yes	Yes	Yes	Yes	Yes	Yes	Yes
Observations	454	25356	25356	25356	22041	25356	25356
R^2^	0.45	0.54	0.54	0.78	0.56	0.54	0.55

#### 4.3.3 Controlling for other policy interference

The level of employee security may be influenced by other policies, such as the development of the digital economy and enterprise digital transformation. First, this study constructs a policy variable for national big data comprehensive pilot zones (*Bigdata*) using the difference-in-differences model (62). Second, a variable for enterprise digital transformation (*Digital*) is constructed through text analysis and logged after adding 1 (63). To control for the effects of digital economy development and enterprise digital transformation, these factors are included as control variables in the baseline regression model.

As shown in Columns (2) and (3) of Table 4, the coefficients of the interaction term (*Treat × Time*) are 1.584 and 1.577, respectively, both significant at the 0.01 level, demonstrating the robustness of the results.

#### 4.3.4 Alternative measurement methods

Considering that employee employment and compensation are also key factors affecting employee safety and health, this study conducts a robustness test by summing the employee employment score, employee compensation score, and employee security score to measure *Safe2*. The indicators for employee employment and compensation scores are shown in Table 6.

**Table 6:** Employee employment and compensation scoring system.

Variable	Indicator	Measurement method
Employee employment	Number of Employees	Number of employees at year-end (people)
	New Job Positions	(Total number of employees this year—Total number of employees last year)/Total number of employees last year
	Gender Diversity in Management	Number of female executives/Total number of executives
	Job Competitiveness and Career Management	(Union expenses + Employee education expenses)/Total operating income
	Care for Disadvantaged Groups	If the enterprise discloses the use of disabled employees, the value is 1; if not disclosed or no disabled employees are used, the value is 0
Employee compensation	Per Share Contribution to Employee Compensation	(Ending balance of payable employee compensation—Beginning balance of payable employee compensation + cash payments made to employees)/Paid-in capital (or equity capital)
	Employee Profit Sharing	Number of employee shareholders/Total number of employees
	Average Compensation per Employee	Payable employee compensation/Number of employees
	Employee Compensation Growth Rate	(Average compensation this year—Average compensation last year)/Average compensation last year
	Ratio of Executive to Other Employees’ Average Compensation	Average annual compensation of directors, supervisors, and executives/Average annual compensation of other employees. The average annual compensation of directors, supervisors, and executives = Total annual compensation of executives/Total number of executives; Average compensation of other employees = (Total employee compensation—Total annual compensation of executives)/(Total number of employees—Total number of executives)

As shown in Column (4) of Table 5, the coefficient of the interaction term (*Treat × Time*) is 3.153, significant at the 0.01 level, confirming the robustness of the research results.

#### 4.3.5 Other robustness tests

To further ensure the robustness of the study, additional tests were conducted. First, the policy interaction term (*Treat × Time*) and all control variables were lagged by one period. Second, to further eliminate potential model specification errors, standard errors were clustered at the enterprise level. Finally, to mitigate the impact of outliers on the analysis, all continuous variables were winsorized at the 1 and 99% levels.

As shown in Columns (5) to (7) of Table 5, the coefficients of the interaction term (*Treat × Time*) remain significantly positive, confirming the robustness of the research results.

## 5 Further analysis

### 5.1 Mechanism test

To examine the mechanisms through which industry-education integrated enterprises enhance employee safety and health levels, this study conducts a mechanism test using a mediation effect model. Table 7 reports the results of the baseline regression based on Equations 2, 3.

First, the natural logarithm of the internal control index (*IC*) plus 1 is taken, and a regression test is conducted. As shown in Column (1) of Table 7, after controlling for individual and time fixed effects and the control variables, the coefficient of the interaction term (*Treat × Time*) is significantly positive at 0.129 at the 0.05 level. This indicates that the implementation of industry-education integration in pilot enterprises has significantly improved the level of internal control.

**Table 7:** Mechanism test.

	*IC*	*Media*	*Safe1*
	(1)	(2)	(3)
*Treat × Time*	0.22^**^	0.33^***^	1.55^***^
	(0.09)	(0.10)	(0.59)
*IC*			0.07^***^
			(0.02)
*Media*			0.07^**^
			(0.03)
_cons	1.17	-1.21^*^	41.53^***^
	(1.78)	(0.71)	(2.96)
Controls	Yes	Yes	Yes
Individual FE	Yes	Yes	Yes
Year FE	Yes	Yes	Yes
Observations	25356	25356	25356
R^2^	0.28	0.84	0.54

Second, the natural logarithm of the number of newspaper mentions plus 1 (*Media*) is taken, and a regression test is conducted. As shown in Column (2) of Table 7, after controlling for individual and time fixed effects and the control variables, the coefficient of the interaction term (*Treat × Time*) is significantly positive at 0.331 at the 0.01 level. This suggests that, following the implementation of industry-education integration, the attention received by pilot enterprises in newspaper coverage significantly increased, indicating an improvement in external supervision.

Finally, *IC* and *Media* are added as control variables to the baseline regression model, and a regression test is conducted. As shown in Column (3) of Table 7, the coefficients of both *IC* and *Media* are significantly positive, indicating that enhancing internal control and external supervision helps improve employee security. Meanwhile, the coefficient of the interaction term (*Treat × Time*) is lower than that in the baseline regression [see Column (2) of Table 4], suggesting that the enhancement of internal control and external supervision is the mechanism through which industry-education integrated enterprises improve employee safety and health levels. Thus, Hypothesis 2 and 3 is confirmed.

### 5.2 Heterogeneity analysis

#### 5.2.1 Nature of ownership

Compared to non-state-owned enterprises, state-owned enterprises (SOEs) not only maintain regular business operations but also fulfill broader responsibilities, such as promoting employment and maintaining social stability. As a result, SOEs may place greater emphasis on employee safety and health. Therefore, the effect of industry-education integration on enhancing employee safety and health levels may be more pronounced in SOEs compared to non-SOEs. Specifically, this study divides the sample into non-SOEs and SOEs based on ownership and conducts a group regression analysis.

As shown in Column (1) and Column (2) of Table 8, the coefficient of the interaction term (*Treat × Time*) is not significant in the non-SOEs group, whereas it is significantly positive in the SOEs group. This indicates that industry-education integration has a more significant effect on improving employee safety and health levels in SOEs.

**Table 8:** Heterogeneity analysis.

	Ownership nature		Management capability	
	(1) Non-SOEs	(2) SOEs	(3) Strong capability	(4) Weak capability
*Treat × Time*	1.29	1.45^*^	2.33^**^	0.65
	(0.87)	(0.80)	(0.98)	(0.68)
_cons	39.08^***^	39.72^***^	43.28^***^	42.41^***^
	(3.87)	(5.72)	(5.48)	(3.44)
Controls	Yes	Yes	Yes	Yes
Individual FE	Yes	Yes	Yes	Yes
Year FE	Yes	Yes	Yes	Yes
Observations	16125	9132	12394	12422
R^2^	0.56	0.53	0.56	0.61

#### 5.2.2 Management capability

Management capability affects the level of internal control. The stronger the management capability, the higher the management efficiency, and the less likely the management will engage in opportunistic behavior through activities such as travel expenses. Therefore, enterprises with stronger management capabilities are likely to place more emphasis on employee safety and health. Specifically, this study uses the management expense ratio as a measure of management capability. A lower management expense ratio indicates that the enterprise incurs fewer management expenses to generate one unit of operating revenue, reflecting higher management capability. The sample is divided into two groups, strong and weak management capability, based on the median of the current year, and a group regression analysis is conducted.

As shown in Column (3) and Column (4) of Table 8, the coefficient of the interaction term (*Treat × Time*) is not significant in the group with weak management capability, while it is significantly positive in the group with strong management capability. This suggests that industry-education integration more significantly enhances employee safety and health levels in enterprises with stronger management capabilities.

### 5.3 Extended analysis

As industry-education integrated enterprises continuously improve employee security, these pilot enterprises become increasingly attractive to highly educated and innovative talent. From a talent agglomeration perspective, the concentration of skilled individuals fosters knowledge spillovers, collaborative learning, and cross-disciplinary innovation (64). The resulting density of human capital enhances R&D efficiency and technological breakthroughs, driving innovation-enhancing benefits.

Concurrently, the industry effect amplifies this dynamic. As talent clusters in these enterprises, they stimulate upstream and downstream linkages, encouraging specialization and economies of scale (65, 66). The presence of high-skilled workers attracts complementary investments, such as advanced infrastructure or specialized suppliers, further reinforcing innovation capacity. Together, the dual mechanisms of talent agglomeration and industrial synergy create a self-reinforcing cycle, positioning industry-education integrated firms as hubs for sustained innovation.

To test whether the implementation of industry-education integration in pilot enterprises promotes enterprise innovation, this study first constructs an OLS regression model controlling for multidimensional fixed effects to examine the impact of the interaction term (*Treat × Time*) on the enterprise’s R&D intensity (*R&D*). Second, a Poisson distribution regression model with multidimensional fixed effects is constructed to test the impact of the interaction term (*Treat × Time*) on the number of patent applications (*Patent*).

Meanwhile, considering that the impact of industry-education integration on enterprise innovation may be disturbed by more potential factors, this paper incorporates additional new control variables in the model. First, this paper controls the characteristics of senior executives, including the proportion of male executives and the average age (67). Second, this paper controls the corporate governance structure, including the nature of property rights and the integration of the two positions. Furthermore, this paper also lags behind the explanatory variables and all control variables by one period (73).

As shown in Table 9, after controlling for individual and time fixed effects, as well as control variables, the interaction term (*Treat × Time*) has a significant positive impact on both R&D intensity [see Column (1)] and the number of patent applications [see Column (2)]. After lagging behind by one period, the promoting effect of industry-education integration on innovation remains unchanged [see Column (3) and (4)]. These results indicate that industry-education integration has an innovation-promoting effect on pilot enterprises, driving both the input and output of innovation resources.

**Table 9:** Extended analysis.

	Current period		Lag by one period	
	(1) *R&D*	(2) *Patent*	(3) *R&D*	(4) *Patent*
*Treat × Time*	0.02^***^	0.12^*^	0.01^***^	0.15^**^
	(0.00)	(0.06)	(0.00)	(0.07)
*gender*	-0.00	0.20	-0.01	0.01
	(0.00)	(0.22)	(0.00)	(0.26)
*age*	0.01^*^	0.24	0.01^**^	-0.15
	(0.01)	(0.34)	(0.01)	(0.41)
*soe*	-0.00^*^	-0.06	-0.00^*^	-0.02
	(0.00)	(0.05)	(0.00)	(0.06)
*dual*	0.00	-0.03	0.00	-0.04
	(0.00)	(0.04)	(0.00)	(0.05)
_cons	-0.10^***^	-7.38^***^	-0.18^***^	1.13
	(0.04)	(2.00)	(0.04)	(2.42)
Controls	Yes	Yes	Yes	Yes
Individual FE	Yes	Yes	Yes	Yes
Year FE	Yes	Yes	Yes	Yes
Observations	25356	24480	20861	20088
R^2^	0.70		0.71	

## 6 Discussions and conclusions

### 6.1 Recommendations

#### 6.1.1 Policy recommendations

The following policy recommendations are proposed, first, on the basis of the initial results achieved by the pilot enterprises in the integration of industry and education, it is necessary to summarise the successful experiences and constraints in a timely manner, gradually expand the scope of the pilot programme and improve this new education model. The government should build a multi-level policy incentive system, centred on subsidies, tax exemptions and financial support, to reduce the cost and risk of enterprises participating in the integration of education and industry. At the same time, the government should strengthen the publicity and interpretation of the policy, so that enterprises and colleges and universities can fully understand the policy dividends, and guide more main bodies to actively participate in the promotion of the integration of industry and education mode of continuous improvement.

Second, the government can clarify the boundaries between the rights and responsibilities of enterprises and institutions through special legislation on the industry-education integration, and stipulate the mandatory obligations and rights and interests of enterprises to participate in the training of talents. Specifically, the government can adopt special legislation to establish the legal status change from student to employee for trainees in the industry-education integration, and mandatorily cover the baseline standards for work injury insurance, annual safety training and internship allowances. Through legislation, the vacuum of the identity of trainees as non-workers and non-students in the industry-education integration can be solved from the source, providing a legal cornerstone for the protection of the rights and interests of quasi-employees.

Finally, as an innovation ecological architect, the government needs to take the lead in building a research and transformation platform driven by the integration of industry and education. The government should take the lead in building a closed-loop ecosystem of industry-education integration based on the principles of demand-led R&D, practical training of talents, and agile transformation of results, so that it can become the core engine of industrial upgrading. Specifically, developed countries can set up technology pioneer funds to subsidise industry-education consortia to carry out scientific research and transformation of results in strategic areas such as artificial intelligence and new energy. Developing emerging economies can selectively build an innovation ecosystem for industry-education integration according to their comparative advantages, and gradually converge with developed countries.

#### 6.1.2 Management recommendations

Based on the research conclusions, the following management implications are obtained in this paper. First, enterprises should actively participate in government-led fusion project production and education, to solve the contradiction between enterprise talent shortage and capacity mismatch, make the transition from attracting talent to retain talent. Enterprises can break down core technology research and development projects into teaching modules, attract trainees to deeply participate in the research and development, and automatically trigger the rights of potential employees through the accumulation of credits, that is, to attract talents. In addition, enterprises can establish an innovation achievement sharing mechanism to share the future benefits of innovation achievements with potential employees, that is, to retain talents.

Second, enterprises should fully support the construction and implementation of teaching fusion, fully enjoy the dividend policy, precise cultivating technical talents, reconstruct enterprise sustainable competitiveness. Enterprises need to increase investment in the integration of industry and education, and regard it as the cost of trial and error in research and development, establishing a mechanism where problems serve as teaching materials. In this way, enterprises can not only cultivate talents but also provide solutions for production practice.

### 6.2 Limitations

However, this study has several limitations. First, the exploration of the underlying mechanisms mainly focuses on internal and external governance perspectives, while other potential mechanisms remain unexplored.

Second, further research is needed to examine other impacts of the pilot construction of industry-education integrated enterprises, such as enterprise productivity and financial performance.

### 6.3 Conclusion

The principal findings of this study are as follows. First, the implementation of industry education integration significantly enhances employee safety and health. This result accords with Thomas et al. (68), who, in their analysis of the triple helix collaborative mechanism, show that collaboration between corporations and universities systematically strengthens occupational safety regimes, reduces workplace injuries, and lowers health losses, thereby confirming the positive role of cross boundary cooperation in improving workplace safety. The present evidence extends these insights to the employee level by documenting additional spillover effects of industry education integration on organizational improvement.

Second, industry education integration elevates employee safety and health assurance by reinforcing both internal control capacity and external institutional oversight. On the one hand, corporations involved in joint curriculum design, training base operation, and joint assessment mechanisms must establish more rigorous managerial rules and process controls, leading to higher quality internal control systems (69, 70). On the other hand, as policy supported pilots, industry education integration corporations are often subject to a double evaluation framework imposed by governmental education regulators and sectoral authorities, which intensifies institutional external monitoring. Strengthened governance mechanisms not only standardize production and operation but also indirectly foster the institutionalisation and routinisation of occupational health management.

Third, the positive effect of industry education integration is more pronounced in state owned corporations and in corporations with stronger managerial capabilities. This finding aligns with Lin and Guan (71), who observe that in state owned corporations, where organizational rigidity and reputation sensitivity are greater, institutional cooperation mechanisms more readily trigger a chain reaction of internal control improvement and employee protection enhancement. This evidence confirms that organizational attributes differentiate the effectiveness of industry education integration.

Fourth, industry education integration markedly boosts corporate innovation capacity, as evidenced by simultaneous increases in the input and output of innovation resources. This result corresponds with Hughes and Kitson (72), who report that university industry collaboration promotes knowledge transfer and innovation generation. It also resonates with employee driven innovation theory, insofar as industry education integration is often accompanied by skill upgrading and greater participation by frontline employees, thereby providing fertile ground for an endogenous innovation culture and practice.

## Data Availability

The raw data supporting the conclusions of this article will be made available by the authors, without undue reservation.
